# Toll-like receptor-2 regulates macrophage polarization induced by excretory-secretory antigens from *Schistosoma japonicum* eggs and promotes liver pathology in murine schistosomiasis

**DOI:** 10.1371/journal.pntd.0007000

**Published:** 2018-12-27

**Authors:** Wenci Gong, Fengjuan Huang, Lei Sun, Aiping Yu, Xiaofan Zhang, Yuxin Xu, Yujuan Shen, Jianping Cao

**Affiliations:** 1 National Institute of Parasitic Diseases, Chinese Center for Disease Control and Prevention; Key Laboratory of Parasite and Vector Biology, Ministry of Health, China; National Center for International Research on Tropical Diseases, China; WHO Collaborating Center for Tropical Diseases, Shanghai, China; 2 Department of Immunology, Tongji University School of Medicine, Shanghai, China; University of Edinburgh, UNITED KINGDOM

## Abstract

Schistosomiasis is endemic to many regions of the world and affects approximately 200 million people. Conventional adaptive T cell responses are considered to be the primary contributors to the pathogenesis of *Schistosoma japonicum* infection, leading to liver granuloma and fibrosis. However, the functional polarization of macrophages and the associated underlying molecular mechanisms during the pathogenesis of schistosomiasis remains unknown. In the present study, we found that excretory-secretory (ES) antigens derived from *S*. *japonicum* eggs can activate macrophages, which exhibit an M2b polarization. Furthermore, ES antigen-induced M2b polarization was found to be dependent on enhanced NF-κB signaling mediated by the MyD88/MAPK pathway in a TLR2-dependent manner. In addition, the cytokine profile of the liver macrophages from wild-type-infected mice are quite distinct from those found in TLR2 knockout-infected mice by quantitative PCR analysis. More importantly, the size of granuloma and the severity of the fibrosis in the livers of TLR2^-/-^ mice were significantly reduced compared to that in WT mice. Our findings reveal a novel role for M2b polarization in the pathogenesis of schistosome infection.

## Author summary

Schistosomiasis is a global health concern that affects primarily tropical and subtropical areas. During a schistosome infection, the eggs are trapped in the host liver and products derived from eggs induce a polarized Th2 response, resulting in granuloma formation and eventually fibrosis. Thus, it is important to elucidate the mechanism of granuloma formation and fibrosis development. Here, we show that activated macrophages play a novel role in the promotion of hepatic granuloma formation and liver fibrosis in a *Schistosoma japonicum*-infected mouse model. In addition, M2b polarization induced by egg products was dependent on enhanced NF-κB signaling mediated by the MyD88/MAPK pathway in a TLR2-dependent manner. Our findings reveal a novel role and mechanism of M2b polarization in the liver pathogenesis in *S*. *japonicum-*infected mice.

## Introduction

Schistosomiasis is one of the most important health problems in developing countries[1], and can be used as a chronic disease model for investigating the interplay between the immune response and parasite pathogenicity in the host[2]. Following a schistosome infection, the host immune response gradually switches from a predominant Th1 response to a Th2-dominated response following egg deposition [3]. The resulting Th2 cytokine secretion contributes to the development of hepatic fibrosis and portal hypertension[4, 5]. A lipid fraction from *Schistosoma mansoni* eggs containing lysophosphatidylserine (lyso-PS) has been shown to induce dendritic cell (DC) activation that promotes Th2 and regulatory T-cell development via a Toll-like receptor (TLR)2-dependent mechanism[6]. Moreover, soluble *S*. *japonicum* egg antigens (SjEA) can upregulate programmed death ligand 2 (PD-L2) expression on bone marrow-derived dendritic cells (BMDCs) in a TLR2-dependent manner to help inhibit T cell responses in *S*. *japonicum* infected mice[2]. More importantly, these data indicate that interactions between host TLRs and pathogen-associated molecular patterns (PAMPs) from schistosome eggs can initiate a Th2-biased immune response and contribute to the egg-induced immunopathology observed in schistosomiasis.

Specialized pattern recognition receptors (PRRs) that recognize PAMPs, and the activation of such PRRs leads to an immediate innate immune response to infection and can profoundly influence the development of an adaptive immune response[7]. Among these PRRs, TLRs are type-1 transmembrane glycoproteins that can identify particular PAMPs and danger associated molecular patterns (DAMPs)[8]. TLRs are well-known to defend against pathogen invasion by triggering innate immune responses and subsequently priming adaptive immunity against infections, including Gram-positive and negative bacteria, as well as fungi, viruses, and parasites[9]. However, some TLRs can trigger a suppressive immune response through the binding of various ligands, which can help avoid excessive inflammation and develop chronic course of the disease, especially in helminth infections[10]. TLRs are expressed on various immune cells, including T cells, B cells, dendritic cells (DCs), and macrophages[11]. TLR engagement results in the activation of the mitogen-activated protein kinases (MAPKs), which, together with the NF-κB pathway, induce extracellular signaling to initiate specific cellular responses[12].

Macrophages, the most abundant mononuclear phagocytes in the human body, are heterogeneous, versatile cells that can undergo dynamic switches in phenotype or function in response to microenvironmental signals[13]. Functional macrophage polarization represents different extremes of a continuum ranging from M1, M2a, and M2b to M2c [14], which can cause different cell populations to display differential gene expression and distinct functions [15]. M1 polarization, driven by IFN-γ and LPS, typically acquires fortified cytotoxic and antitumoral properties, whereas M2 polarization generally obtains immunoregulatory activities, tissue repair, and remodeling[16]. In particular, M2a polarization is induced by IL-4 and IL-13, whereas M2b polarization, induced by immune complexes and TLR or IL-1R agonists, is characterized by an IL-10^high^ and IL-12^low^ phenotype, exerts immunoregulatory functions, and drives Th2 responses. In contrast, M2c polarization, induced by IL-10 and glucocorticoid hormones, results in immunosuppression and tissue-remodeling activities[14]. Although the critical role of macrophage activation in the pathogenesis of schistosomiasis has been validated[17], the precise phenotype and mechanism associated with functional macrophage polarization in schistosomiasis remains unclear.

In this study, we identified a novel role for macrophages in liver pathogenesis using a *S*. *japonicum*-infected mouse model and present TLR2 signaling as a novel potential therapeutic target for schistosomiasis.

## Methods

### Ethics statement

All animal experiments were performed in strict accordance with the Regulations for the Administration of Affairs Concerning Experimental Animals (approved by the State Council of People’s Republic of China), and efforts were made to minimize suffering. All procedures performed on animals in this study were approved by the Laboratory Animal Welfare & Ethics Committee (LAWEC) of National Institute of Parasitic Diseases (Permit Number: IPD-2016-7).

### Mice and parasites

Female C57BL/6 mice (6- to 8-weeks-old) were purchased from the SLAC laboratory (Shanghai, China). TLR2^-/-^ mice[18] were provided by Dr. Xiao-Ping Chen from the School of Medicine, Tongji University. All mice were maintained under specific pathogen-free conditions and fed with standard laboratory food and water. Gender and age-matched mice were infected percutaneously with 20 ± 1 cercariae of *S*. *japonicum*, which were shed from infected *Oncomelania hupensis* snails provided by the National Institute of Parasitic Diseases in Shanghai, China.

### Preparation and treatment of excretory-secretory (ES) antigens

*S*. *japonicum* eggs were isolated from the livers of female rabbits 6 weeks following infection with 800 − 1000 cercariae via abdominal skin penetration. ES antigens were prepared as described previously with modifications[19]. The collected eggs were washed twice in serum-free DMEM supplemented with 100 U/mL penicillin and 100 μg/mL streptomycin. The eggs were then resuspended in 24 mL DMEM, and 2 mL aliquots were placed in six-well culture plates (Corning, USA). The culture medium was harvested after 48 h and centrifuged for 10 min at 200 × *g* to remove eggs, and 10000 × *g* to remove any debris. The protein concentration of ES antigens was determined using a Bradford assay. The endotoxin level of ES antigens was <0.03 EU/mL as determined by a *Limulus amoebocyte lysate* assay (Genscript, China) according to the manufacturer’s instructions. ES antigens were stored at -80°C until further use.

To destroy the lipid structures, ES antigens were digested with phospholipase C (Sigma, USA) at 37°C for 12 h, followed by heat inactivation of the enzymes at 100°C for 10 min. To digest proteins, ES was treated with proteinase K (Sigma, USA) at 56°C overnight, followed by heat inactivation of the enzymes at 100°C for 10 min. Mock-treated ES was also performed by heat at 100°C for 10 min without the addition of enzymes. Protein disruption was regularly checked by SDS-PAGE and viewed by silver staining.

### Bone-marrow-derived macrophage (BMDM) preparation

BMDMs were prepared as previously described with modifications[20]. Briefly, bone marrow cells were isolated from the leg bones of wild-type and TLR2^-/-^ mice and cultured in DMEM (Gibco, USA) supplemented with 10% FBS (Gibco, USA) and 50 ng/mL macrophage colony-stimulating factor (M-CSF) (Peprotech, USA) and maintained in a 5% CO_2_ incubator at 37°C. Six days after the initial BM cell culture, the medium was changed, and the purity of F4/80^+^ cells was > 99%, as determined by flow cytometry.

In some experiments, BMDM cells (5 × 10^5^ cells/mL) were pretreated with one of the following inhibitors: 10 μM BAY 11–7082 (NF-κB inhibitor, Beyotime biotechnoogy, China), 10 μM SP 600125 (JNK MAPK inhibitor, Beyotime biotechnoogy, China), 1 μM SB 203580 (p38 MAPK inhibitor, Beyotime biotechnology, China), 10 μM PD 98059 (ERK MAPK inhibitor, Beyotime biotechnology, China) and 10 μM ST 2825 (MyD88 homodimerization inhibitor, MedChem Express, USA). Furthermore, LPS from *Escherichia coli* serotype O111:B4 (Sigma, USA) and synthetic lipoprotein Pam3CSK4 (InvivoGen, USA) were used in some experiments.

### Reverse transcription quantitative real-time PCR (RT-qPCR)

The total RNA was extracted from macrophages using Trizol reagent (Invitrogen, USA) and reversed transcribed using a cDNA reverse transcription kit (Takara, Japan). The reverse-transcribed cDNA was used as a template in qPCR reactions containing SYBR Green Real-time PCR Master Mix (Takara, Japan) and 0.4 μM forward and reverse primers. Relative mRNA expression was calculated using the 2-^ΔΔCt^ method and normalized to glyceraldehyde-3-phosphate dehydrogenase (GAPDH). The primer sequences were prepared as previously described[16, 21] and are listed in S1 Table.

### Enzyme-linked immunosorbent assay (ELISA)

BMDMs were stimulated with different antigens for 24 h. The levels of IL-1β, IL-12p70, IL-6, MCP-1 (CCL2), TNF-α, and IL-10 in the supernatants were detected by ELISA (eBioscience, USA). Quantification of IFN-γ, IL-4 and IL-13 in the serum sample was also determined by an ELISA in accordance with the manufacturer’s instructions (eBioscience, USA) and expressed as pg per mL.

### Western blot analysis

Macrophages (5 × 10^5^ cells/mL) were stimulated with various doses of ES (0.1 μg/mL, 1 μg/mL, and 10 μg/mL), LPS (100 ng/mL), and Pam3CSK4 (4 μg/mL) for different time points. The treated cells were washed twice with PBS and then lysed for 30 min on ice in a RIPA solution containing a protease inhibitor cocktail and phosphatase inhibitors (Sigma, USA). The expression of proteins in the cell lysates were examined using anti-NF-κB phospho-p65, anti-phospho-JNK, anti-phospho-p38, and anti-phospho-ERK1/2 antibodies (Cell signaling technology, USA). Anti-GAPDH (Sungene Biotech, China) was used as an internal control. Statistical analysis was performed for band intensities and evaluated using image J (NIH, USA).

### Cell isolation and flow cytometry

The cellular suspension of liver leukocytes was prepared using the traditional method according to previous reported methods with modifications [22]. In brief, following a perfusion of 3 mL PBS via the portal vein, mouse liver fragments were pressed through a 70-μm cell strainer (BD, USA). The total liver cells were then resuspended in a 40% Percoll solution (GE Healthcare, USA), and centrifuged for 20 min at 800 × *g*. The leukocytes were resuspended in an erythrocyte-lysing buffer. The cells were washed and resuspended in a MACS separation buffer (Miltenyi Biotec, Germany), and anti-F4/80 microbeads (Miltenyi Biotec, Germany) were used to isolate macrophages from leukocytes. The purity of the isolated cells was confirmed at > 95%. In some experiments, liver macrophages with a purity of approximately 95% were used as the starting material for ES antigen stimulation. To assess the expression of activation and other biological markers on macrophages, flow cytometry was performed with FITC labeled anti-F4/80, APC labeled anti-Gr-1, Brilliant Violet 421 labeled anti-CD11b, PE labeled anti-MHC class II, PE-Cy7-labeled anti-CD40, PE-Cy7-labeled anti-CD80, APC-labeled anti-CD86, PE-labeled anti-CD16/32 and APC-labeled anti-mannose receptor (CD206) (Biolegend, USA). All flow cytometry data was acquired on an LSRFortessa X-20 (BD Biosciences, USA) and analyzed with FlowJo software (Tree star, USA).

### Histology

Fresh liver tissues were fixed in 4% formaldehyde overnight and routinely paraffin embedded. Paraffin sections (5 μm) were prepared from each liver tissue sample. H&E staining of liver tissue sections were performed according to the manufacturer’s instructions and assessed by a pathologist blinded to the treatment group. The liver tissue sections were stained with Masson’s trichrome staining to evaluate collagen content and distribution. The collagen fibers were stained blue, the cell nuclei were stained black, and the background was stained red. Each stained section was examined by optical microscopy with 100 × magnification and identical settings. Thirty pictures were taken of granulomas around single eggs from three sections in each tissue. Every picture was evaluated in a double-blind fashion by two independent investigators. The area featuring granulomas and fibrosis surrounding single eggs was evaluated using image J (NIH, USA).

### Statistical analysis

Data represented as the mean ± SEM were analyzed by a two-tailed Student’s *t*-test, or a one-way or two-way ANOVA using GraphPad Prism version 5.0 (GraphPad Software, USA). Significant differences were accepted when the *p-*value was less than 0.05.

## Results

### Macrophages treated with ES antigens exhibit an M2b-polarized phenotype *in vitro*

**Fig 1:**
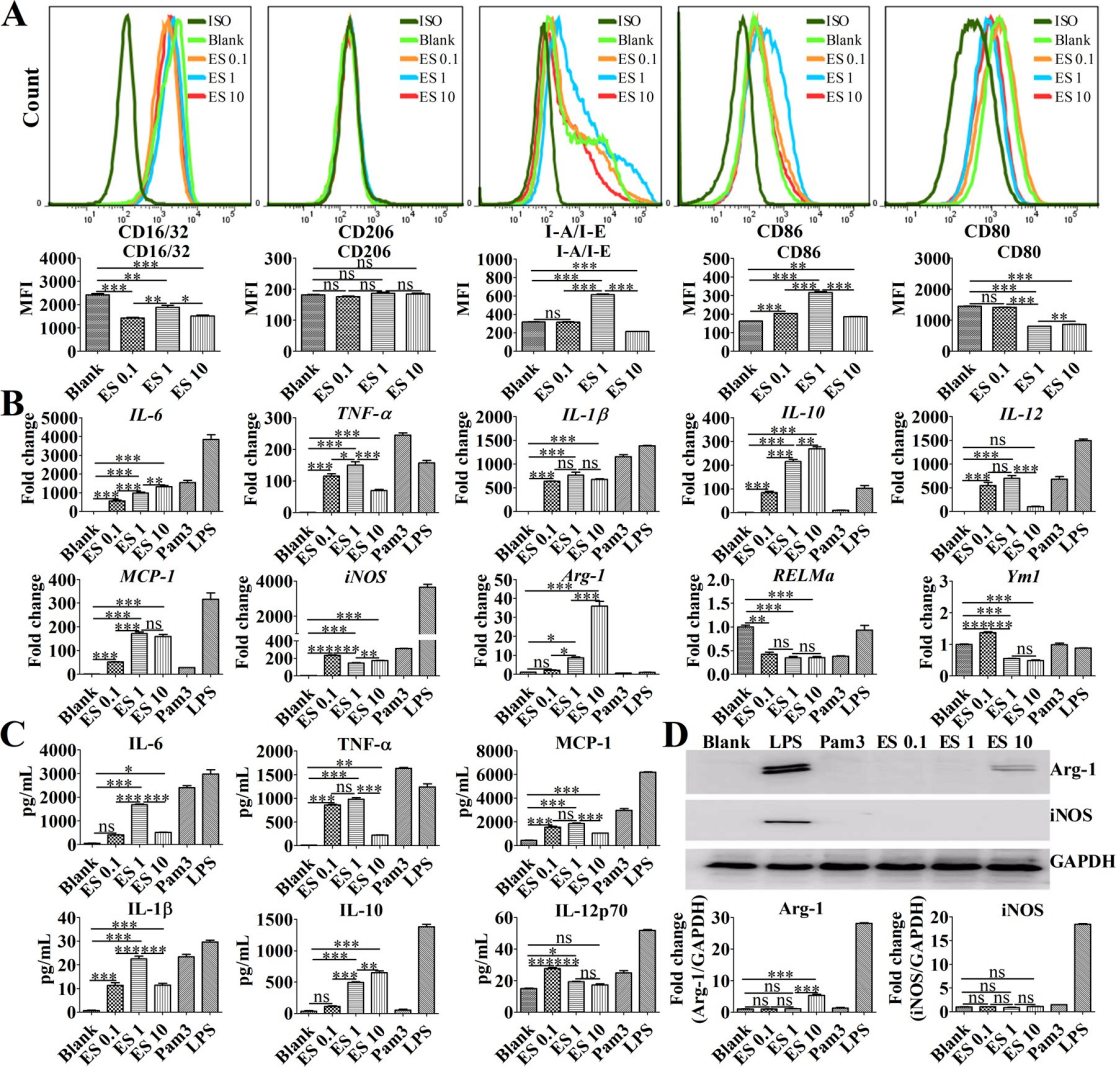
ES induces macrophage M2b polarization *in vitro*. BMDMs were simulated with 0.1 μg/mL ES (ES 0.1), 1 μg/mL ES (ES 1), 10 μg/mL ES (ES10), 100 ng/mL LPS, 4 μg/mL Pam3CSK4 (Pam3), or DMEM. (A) Representative flow cytometry histograms and MFI (median fluorescence intensity) of CD16/32, CD206, MHC class II, CD86, and CD80 expression on BMDMs after stimulation for 24 h. (B) Cellular mRNA levels of *IL-6*, *TNF-a*, *IL-10*, *IL-12*, *IL-1β*, *MCP-1*, *iNOS*, *Arg-1*, *RELMa*, and *Ym1* in BMDMs treated with various antigens or media control for 4 h were analyzed by RT-qPCR. (C) The levels of IL-6, TNF-α, IL-1β, IL-10, IL-12p70, and MCP-1 in supernatants of BMDMs after stimulation for 24 h were measured by an ELISA. (D) The levels of iNOS and Arg-1 protein expression in the lysates of BMDMs after stimulation for 24 h were examined by Western blot. Graphical representations of the band intensities are shown in the pictures below. The expression of iNOS and Arg-1 was normalized to GAPDH expression. The data are the result of a representative experiment out of three independent experiments and analyzed with one-way ANOVA. *P < 0.05; **P < 0.01; ***P < 0.001; ns, not significant.

To confirm that ES stimulation could induce macrophage polarization, BMDMs were stimulated with different concentrations of ES *in vitro*. Enhanced CD86 expression was observed in the BMDMs that were treated with 0.1 μg/mL or 1 μg/mL of ES compared with that of the control group (Fig 1A). However, CD206 (mannose receptor) and CD16/32 expression did not increase after ES stimulation (Fig 1A). Moreover, compared with 0.1 μg/mL ES, stimulation with 1 μg/mL ES was found to upregulate MHC class II (I-A/I-E) and CD86 expression but downregulate CD80 expression. However, compared with 1 μg/mL of ES stimulation, 10 μg/mL of ES stimulation was found to downregulate MHC class II, CD16/32, and CD86 expression (Fig 1A). RT-qPCR analysis showed that BMDMs stimulated with ES antigens exhibited enhanced *IL-6*, *IL-10*, and *Arg-1* mRNA levels in a dose-dependent manner (Fig 1B). Furthermore, 1 μg/mL of ES antigen stimulation increased the levels of *IL-6*, *TNF-α*, *MCP-1*, *IL-10*, and *Arg-1* mRNA expression but decreased the levels of *iNOS* and *Ym1* mRNA compared with 0.1 μg/mL ES antigen stimulation. However, RT-qPCR revealed that the levels of *TNF-α* and *IL-12* mRNA expression were downregulated in BMDMs stimulated with 10 μg/mL of ES antigens compared to BMDMs stimulated with 1 μg/mL of ES antigens (Fig 1B). Similar to these results, significantly higher levels of TNF-α, IL-12p70, MCP-1 (CCL2), and IL-1β were observed in the supernatants of BMDMs stimulated with 0.1 μg/mL ES compared with that of the control macrophages (Fig 1C). Moreover, stimulation with 1 μg/mL ES antigens upregulated IL-6, IL-1β, and IL-10 expression but downregulated IL-12p70 expression compared with 0.1 μg/mL ES antigen stimulation. However, compared with BMDMs stimulated with 1 μg/mL of ES antigens, the levels of IL-1β, TNF-α, MCP-1, and IL-6 were downregulated in the supernatants of BMDMs stimulated with 10 μg/mL ES (Fig 1C). Surprisingly, unlike LPS, ES stimulation did not increase iNOS protein expression but enhanced the production of Arg-1 protein (Fig 1D). According to the reported secretory products and biological surface markers associated with macrophage polarization[16], these data suggest that 1 μg/mL of ES antigen stimulation promotes TNF-α, IL-1β, IL-6, and IL-10, as well as promotes MHC class II and CD86 expression, whereas there are low levels of IL-12 production. These findings indicate that macrophages treated with 1 μg/mL ES display an M2b-polarized phenotype *in vitro*.

### ES antigen stimulation upregulates MAPK/NF-κB signaling in M2b macrophages

**Fig 2:**
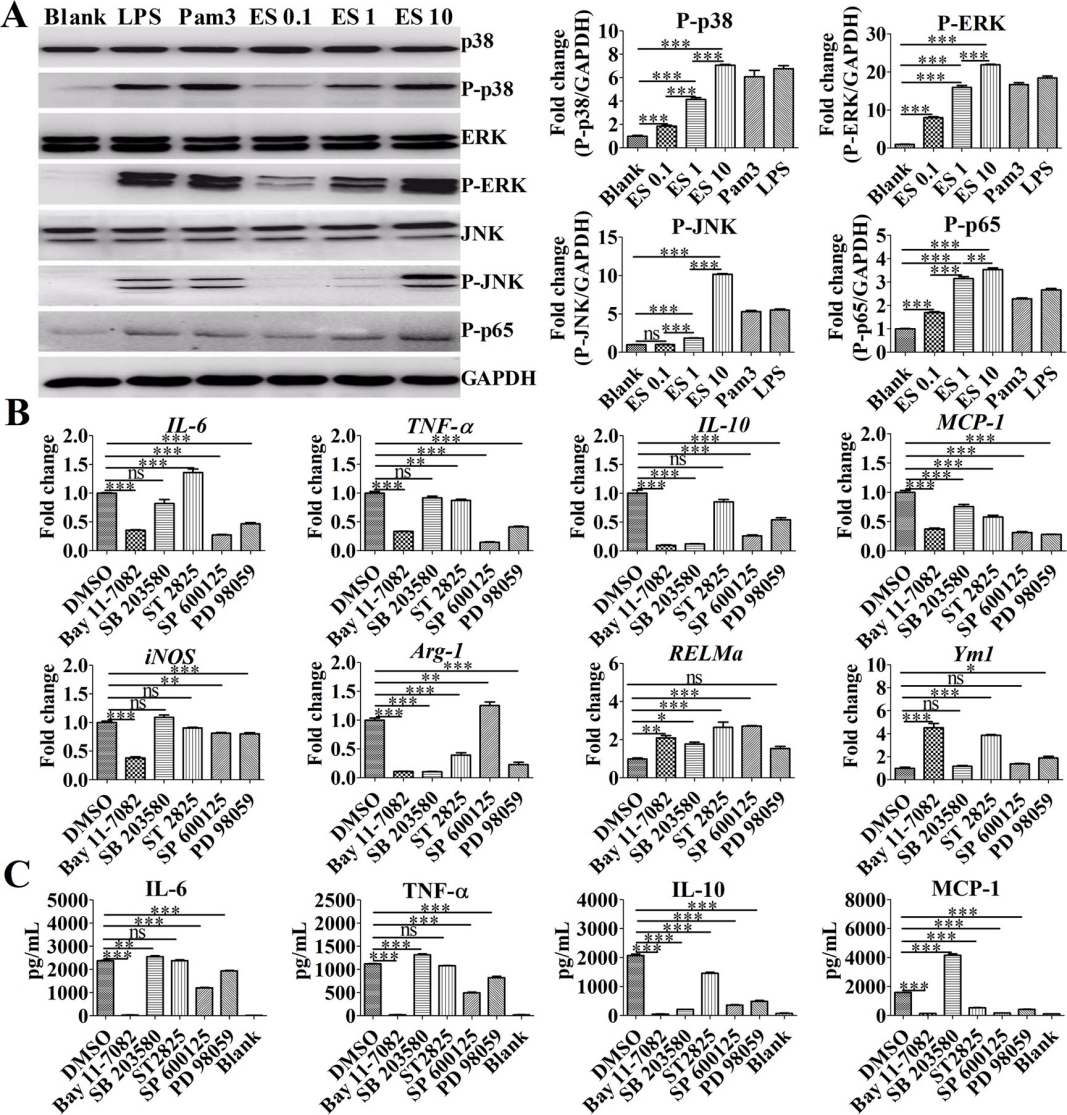
ES promoted M2b polarization and inflammatory cytokine production by activating the MAPK/NF-κB signaling pathway. (A) Immunoblot of P-p38, P-p65, P-ERK1/2, and P-JNK in the lysates of BMDMs after stimulation for 30 min. Graphical representations of band intensities are shown in the right panel. Expression of P-p38, P-p65, P-ERK1/2, and P-JNK was normalized to GAPDH expression. (B) The levels of *IL-6*, *TNF-a*, *IL-10*, *MCP-1*, *Arg-1*, *RELMa*, *Ym1*, and *iNOS* mRNA in BMDMs were analyzed by RT-qPCR. An NF-κB inhibitor (BAY 11–7082, 10 μM), p38 MAPK inhibitor (SB 203580, 1 μM), JNK inhibitor (SP 600125, 10 μM), MyD88 homodimerization inhibitor (ST 2825, 10 μM), or ERK inhibitor (PD 98059, 10 μM) was added to different groups of cells and incubated for 1 h before ES antigen treatment for 4 h. (C) Levels of IL-6, MCP-1, TNF-α, and IL-10 in the supernatants of macrophages stimulated with ES (1 μg/mL) were measured by ELISA. An NF-κB inhibitor (BAY 11–7082, 10 μM), p38 MAPK inhibitor (SB 203580, 1 μM), JNK inhibitor (SP 600125, 10 μM), MyD88 homodimerization inhibitor (ST 2825, 10 μM), or ERK inhibitor (PD 98059, 10 μM) was added to the cells and incubated for 1 h before antigen treatment for 24 h. Data are the results from a representative experiment from three independent experiments and are analyzed by one-way ANOVA. *P < 0.05; **P < 0.01; ***P < 0.001; ns, not significant.

Previous reports indicate that MAPK-NF-κB signaling contributes to macrophage activation[23, 24]. To determine the expression pattern of MAPKs and NF-κB on activated macrophages, a Western blot was performed to analyze the levels of phospho-p38, phospho-p65, phospho-ERK, and phospho-JNK. As shown in Fig 2A, BMDMs treated with ES exhibited increased levels of phospho-p38, phospho-p65, phospho-ERK, and phospho-JNK expression in activated macrophages in a dose-dependent manner *in vitro*. As shown in Fig 2B, the RT-qPCR analysis revealed that BMDMs stimulated with ES antigens exhibited significantly decreased levels of *IL-6*, *TNF-α*, *IL-10*, *MCP-1*, and *iNOS* mRNA expression compared with the control BMDMs upon treatment with PD 98059 (ERK1/2 MAPK inhibitor), SP 600125 (JNK MAPK inhibitor), or BAY 11–7082 (NF-κB inhibitor). However, ST 2825 treatment markedly increased the levels of *IL-6*, *RELMa*, and *Ym1* mRNA but decreased the levels of *TNF-α*, *MCP-1*, and *Arg-1* mRNA compared with control group. Furthermore, SB 203580 (p38 MAPK inhibitor) treatment significantly decreased the levels of *IL-10*, *MCP-1*, and *Arg-1* mRNA but increased the levels of *RELMa* mRNA compared with control BMDMs. Similar to these results, an ELISA was performed to assess the production of inflammatory markers in the supernatants of BMDMs exhibited remarkably decreased production of IL-6, MCP-1, TNF-α, and IL-10 upon treatment with PD 98059 (ERK1/2 MAPK inhibitor), SP 600125 (JNK MAPK inhibitor), or BAY 11–7082 (NF-κB inhibitor) (Fig 2C). However, BMDMs treated with ST 2825 exhibited significantly decreased levels of IL-10 and MCP-1. Furthermore, BMDMs treatment with SB 203580 (p38 MAPK inhibitor) displayed increased levels of IL-6, MCP-1, and TNF-α expression but remarkably decreased levels of IL-10 expression. These data demonstrate that the MyD88/MAPK/NF-κB signaling pathway facilitates ES-induced M2b polarization.

### TLR2 is the pivotal receptor for M2b polarization *in vitro*

**Fig 3:**
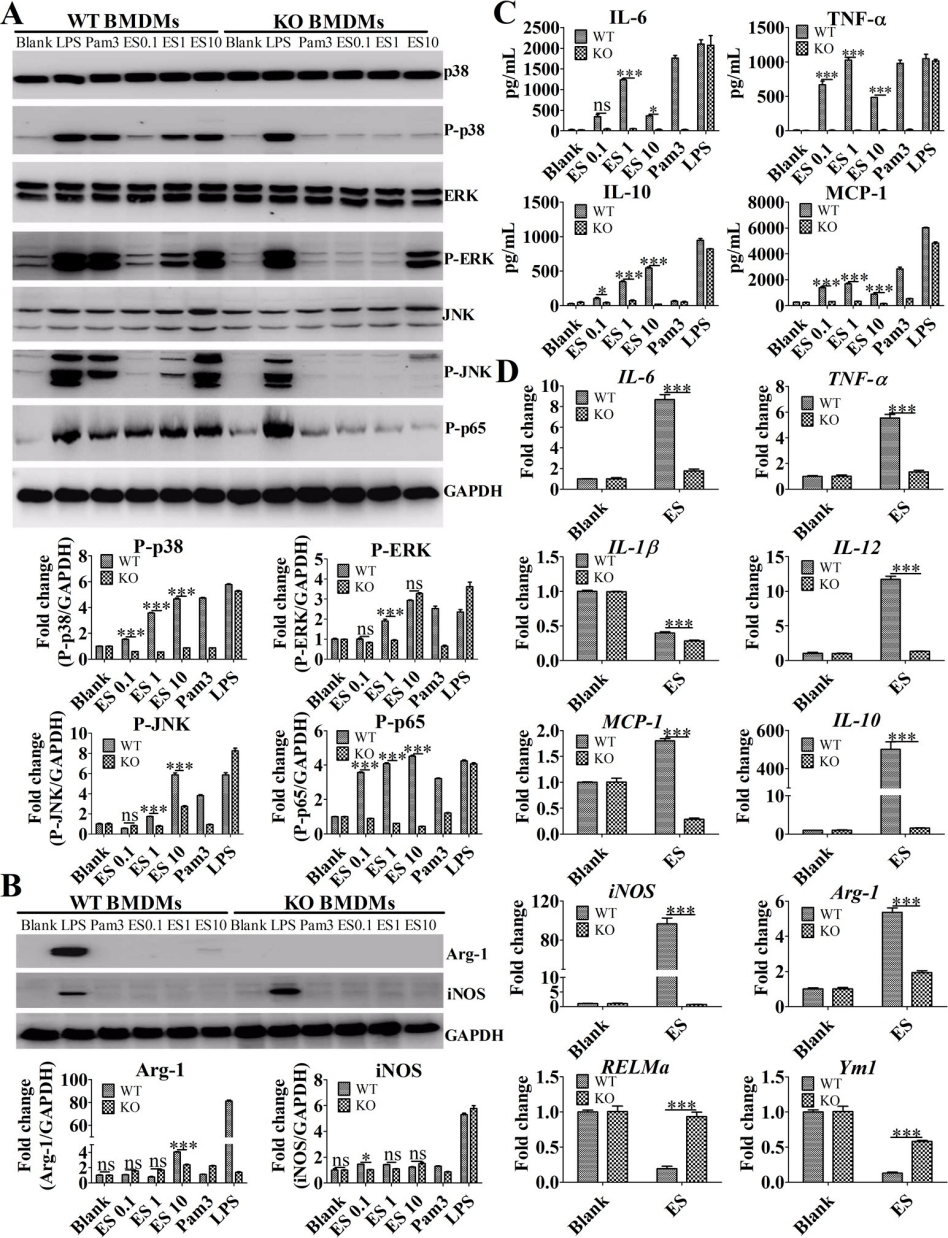
ES promoted M2b polarization by activating the MAPK/NF-κB signaling pathway in a TLR2-dependent manner. (A) Immunoblot of P-p38, P-p65, P-ERK1/2, and P-JNK in the lysates of BMDMs after stimulation for 30 min. Graphical representations of the band intensities are shown in the pictures below. Expression of P-p38, P-p65, P-ERK1/2, and P-JNK was normalized to GAPDH expression. (B) The expression of iNOS and Arg-1 protein in the lysates of BMDMs following stimulation for 24 h was assessed by Western blot. Graphical representations of the band intensities are shown in the pictures below. Expression of iNOS and Arg-1 was also normalized to GAPDH expression. (C) The levels of IL-6, MCP-1, TNF-α, and IL-10 in the supernatants of BMDMs were measured by ELISA. (D) The levels of *IL-6*, *TNF-a*, *IL-10*, *IL-12*, *IL-1β*, *MCP-1*, *iNOS*, *Arg-1*, *RELMa*, and *Ym1* mRNA in the purified liver macrophages from wild-type and TLR2 KO mice after stimulation for 4 h were analyzed by RT-qPCR. The data were expressed as the mean ± SEM, and are the results of a representative experiment out of three independent experiments and analyzed by two-way ANOVA. *P < 0.05; **P < 0.01; ***P < 0.001; ns, not significant.

Multiple previous studies have reported TLR2 to be an important PPR for soluble egg antigens (SEA) [2,25]. To further evaluate the role of the TLR2 receptor on macrophage M2b polarization induced by ES, BMDMs derived from wild-type and TLR2 knockout (KO) mice were stimulated with ES *in vitro*. As shown in Fig 3A, compared with the BMDMs from wild-type mice, BMDMs derived from TLR2 KO mice stimulated with ES did not exhibit increased protein levels of phospho-p38, phospho-p65, phospho-ERK, and phospho-JNK in a dose-dependent manner. However, BMDMs derived from TLR2 KO mice stimulated with a high dose of ES could increase the levels of phospho-ERK protein expression. Treatment BMDMs derived from TLR2 KO mice with ES failed to induce Arg-1 production (Fig 3B). Compared with that of BMDMs from wild-type mice, the ELISA analysis for inflammatory marker production in the supernatants of BMDMs from TLR2 KO mice showed that the levels of IL-6, MCP-1, TNF-α, and IL-10 markedly decreased (Fig 3C). More importantly, RT-qPCR analysis for inflammatory gene expression showed that liver macrophages purified from wild-type mice stimulated with 1 μg/mL of ES exhibited enhanced *IL-10*, *TNF-a*, *IL-1β*, *IL-6*, *IL-12*, *MCP-1*, *Arg-1*, and *iNOS* but reduced the *RELMa* and *Ym-1* mRNA levels compared with liver macrophages purified from TLR2 KO mice (Fig 3D). This indicates that similar to BMDMs (S1 Fig), liver macrophages can be activated by ES antigens in a TLR2-dependent manner. Our results show that TLR2 is the pivotal receptor for M2b polarization following ES antigen stimulation.

### Phospholipase C treatment blunts ES-induced M2b polarization

**Fig 4:**
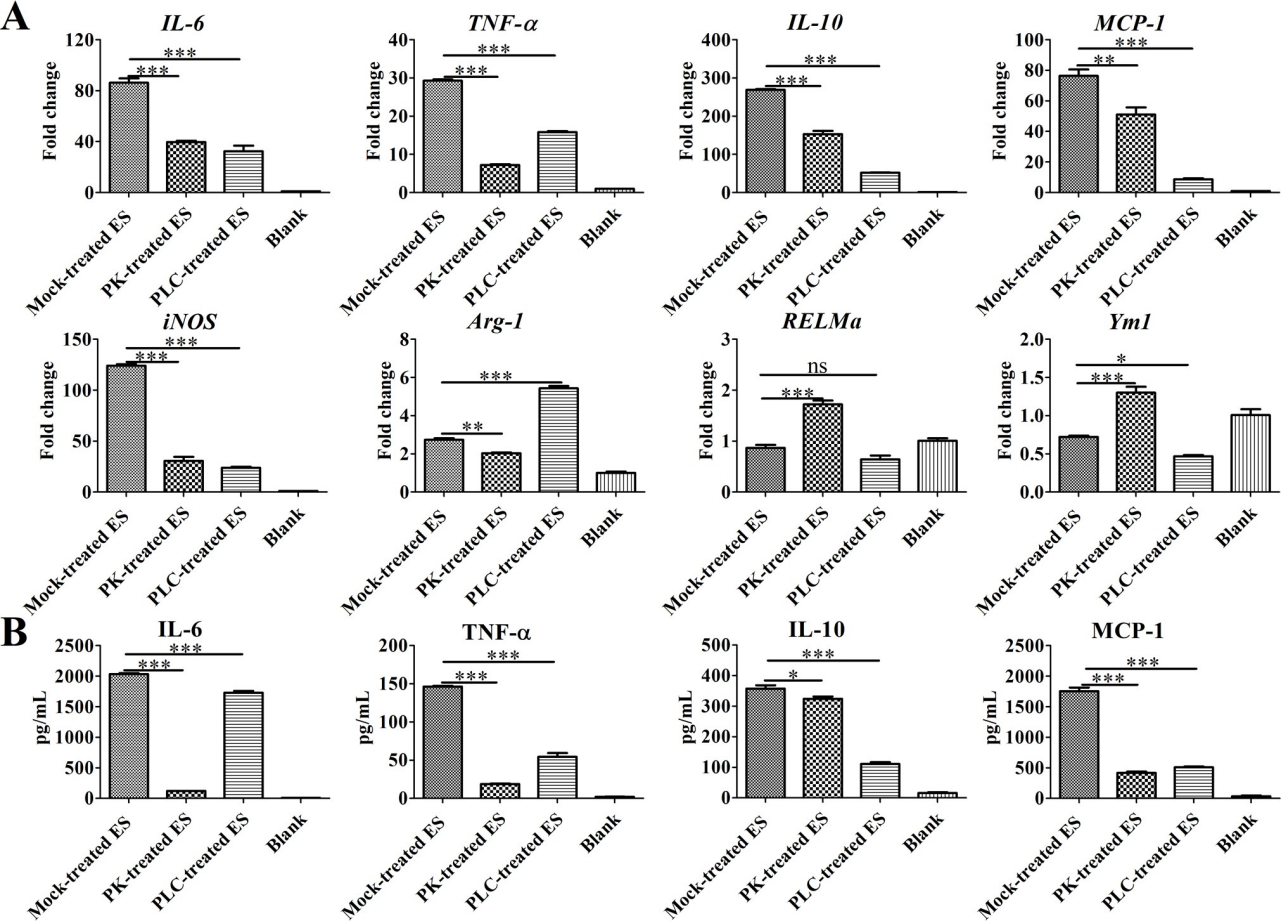
Phospholipase C treatment blunts ES-induced M2b polarization. (A) The levels of *IL-6*, *TNF-a*, *IL-10*, *MCP-1*, *Arg-1*, *RELMa*, *Ym1*, and *iNOS* mRNA in BMDMs stimulated with mock-treated ES (1 μg/mL), proteinase K (PK)-treated ES, phospholipase C (PLC)-treated ES or media control for 4 h were analyzed by RT-qPCR. (B) The levels of IL-6, TNF-α, IL-10, and MCP-1 in the supernatants of macrophages stimulated with mock-treated ES (1 μg/mL), proteinase K (PK)-treated ES, phospholipase C (PLC)-treated ES or media control for 24 h were measured by ELISA. Data, shown as the mean ± SEM, are the results of a representative experiment out of three independent experiments and analyzed by one-way ANOVA. *P < 0.05; **P < 0.01; ***P < 0.001; ns, not significant.

Previous studies have reported schistosome-specific lysophosphatidylcholine (lyso-PS) in SEA activated TLR2[6, 26]. To clarify whether the lipids in ES contribute to the production of pro-inflammatory cytokines by macrophages, RT-qPCR was used to assess the levels of *IL-6*, *TNF-α*, *IL-10*, *MCP-1*, and *iNOS* mRNA, which were found to be decreased in BMDMs stimulated with both proteinase K-treated ES and phospholipase C-treated ES, compared with mock-treated ES (Fig 4A). However, phospholipase C-treated ES treatment increased the levels of *Arg-1* mRNA but decreased *Ym1* mRNA in BMDMs compared with mock-treated ES. Furthermore, proteinase K-treated ES enhanced the levels of *RELMa* and *Ym1* mRNA but reduced *Arg-1* mRNA compared with mock-treated ES. Similar to these data, an ELISA was used to further assess the levels of TNF-α, IL-6, IL-10, and MCP-1 in the supernatants of BMDMs. As shown in Fig 4B, compared to the mock-treated ES, the proteinase K-treated ES as well as phospholipase C-treated ES failed to induce BMDMs to produce high levels of TNF-α, IL-6, IL-10, and MCP-1. Thus, lipids or lipid conjugates contribute to M2b polarization.

### TLR2 promotes M2 polarization and liver pathology *in vivo*

**Fig 5:**
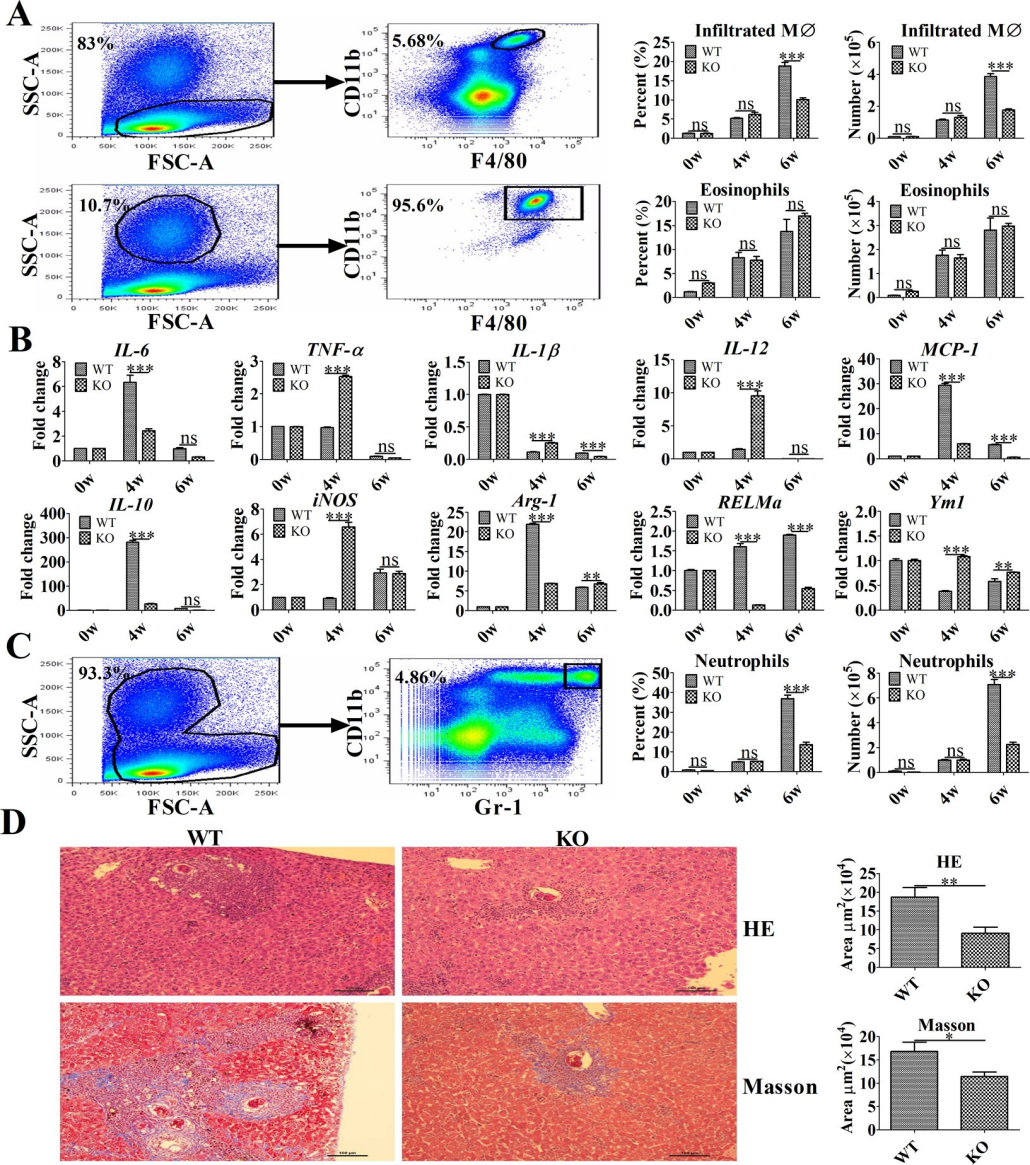
TLR2 promotes M2 polarization and liver pathology in murine schistosomiasis. (A) The absolute numbers of eosinophils and infiltrated macrophages (MØ) in the liver and percentages of eosinophils and infiltrated macrophages (MØ) of the total liver leukocytes were determined by flow cytometry. Representative flow cytometry analysis of infiltrated macrophages (SSC^low^CD11b^+^ F4/80^+^ cells) and eosinophils (SSC^high^CD11b^+^F4/80^+^ cells) among liver leukocytes from one wild-type-infected mouse at 4 weeks post-infection. The data are presented as the mean ± SEM obtained from 10 mice per group and analyzed by two-way ANOVA. (B) The levels of *IL-6*, *MCP-1*, *IL-10*, *IL-12*, *TNF-a*, *IL-1β*, *Arg-1*, *RELMa*, *Ym1*, and *iNOS* mRNA in purified macrophages were analyzed by real-time PCR. The data are the results of a representative experiment out of three independent experiments and analyzed by two-way ANOVA. (C) The absolute number of neutrophils in the liver and percentage of neutrophils among the total liver leukocytes was determined by flow cytometry. Representative flow cytometric analysis of neutrophils (CD11b^+^Gr-1^+^ cells) among liver leukocytes from one wild-type-infected mouse at 4 weeks post-infection. Data are presented as the mean ± SEM obtained from 10 mice per group and analyzed by two-way ANOVA. (D) The area of liver granulomas and fibrosis developed following *S*. *japonicum* infection were detected at 6 weeks post-infection. Slide sections from infected mice were examined under a light microscope, and images were captured and analyzed. Representative granuloma in the liver sections of TLR2^-/-^ and wild-type mice (hematoxylin-eosin [upper panel] and Masson’s trichrome [lower panel]). Original magnification: × 100. Data are presented as the mean ± SEM and were analyzed by Student’s *t*-test. *P < 0.05; **P < 0.01; ***P < 0.001; ns, not significant.

Although previous studies on schistosomiasis often focus on T and B lymphocytes, APCs (e.g., macrophages) may play vital roles in the pathogenesis of the disease [27]. To evaluate the role of macrophages during the pathogenesis of liver granuloma formation and fibrosis in a murine model of schistosomiasis, liver leukocytes were isolated and analyzed for the presence of CD11b^+^F4/80^+^ cells. The absolute number and percentage of eosinophils (SSC^high^CD11b^+^F4/80^+^ cells) and infiltrated macrophages (SSC^low^CD11b^+^F4/80^+^ cells) were substantially increased in the liver issues of the infected mice (Fig 5A). Four weeks post-infection, a real-time PCR analysis of inflammatory gene expression revealed that purified macrophages from infected WT mice exhibited enhanced *IL-10*, *Arg-1*, *MCP-1*, *RELMa*, and *IL-6* but reduced *TNF-α*, *IL-12*, and *iNOS* mRNA levels, which exhibited an M2 dominant- polarized phenotype (Fig 5B). However, compared with WT mice, the purified macrophages from infected TLR2^-/-^ mice exhibited higher levels of *TNF-α*, *IL-12*, and *iNOS* mRNA, as well as lower levels of *IL-10*, *Arg-1*, and *RELMa* mRNA, which represents a dominant M1-polarized phenotype (Fig 5B). Moreover, a significant decrease in the percentage of infiltrated macrophages and neutrophils (CD11b^+^Gr-1^+^ cells) recruitment was observed in infected TLR2^-/-^ mice, compared with that of wild-type mice at 6 weeks post-infection (Fig 5A and Fig 5C). More importantly, the area of granuloma formation and fibrosis surrounding single eggs in the livers of TLR2^-/-^ mice were significantly lower compared with that of WT mice at 6 weeks post-infection (Fig 5D).

## Discussion

Due to their inherent plasticity and heterogeneity, the ability of macrophages to functionally switch from killing pathogens to the promotion of tissue repair is likely critical for the host, especially when the host cannot eradicate a persistent infection but must limit tissue damage (e.g., chronic helminth infection)[17]. The differential activation status of macrophages has the capability to promote, restrict, or resolve inflammation and fibrosis in schistosomiasis [17, 27, 28]. In the present study, we aimed to investigate the phenotypical and functional plasticity of various macrophage subtypes following infection with *S*. *japonicum* (e.g., the presence of ES antigens derived from *S*. *japonicum* eggs). BMDMs stimulated with ES exhibit an activation status characterized by the production of multiple pro-inflammatory cytokines and abundant anti-inflammatory IL-10 production *in vitro*, following the activation of several MAPKs downstream of both TLR2 and MyD88.

Several helminth antigens are known to be associated with the stimulation of various TLRs[29, 30], whereas schistosoma antigens have been reported to interact with specific TLRs present on mononuclear phagocytes[25, 31–33]. However, few of these studies describe the signaling pathways triggered in host cells or link them to the production of specific cytokines[25, 33, 34]. In the current study, we demonstrated that ES-stimulated BMDMs produce pro-inflammatory cytokines and the anti-inflammatory cytokine IL-10 in a dose-dependent manner via TLR2. Moreover, we found that lipids or lipid conjugates participate in macrophage polarization, since the levels of cytokines decreased significantly in the supernatants of BMDMs induced by phospholipase C-treated ES. More importantly, our data confirmed that BMDMs exposed to the ES of eggs were dependent on the phosphorylation of three MAPK cascades (p38, ERK1/2, and JNK1/2), an event often reported downstream of TLR activation[35]. Notably, these kinase cascades occur with identical activation profiles, leading to the phosphorylation of p65 reported to be downstream of p38, ERK1/2, and JNK1/2. However, the full details of these signaling pathways has not previously been reported in the context of ES antigens. Moreover, in the present study, we provide the complete molecular mechanism of specific cytokine production by macrophages in response to ES antigens.

M2 macrophages can express Arg1, an enzyme adapted from the urea cycle which converts L-arginine to L-ornithine, enabling L-ornithine amino-transferase (OAT) to supply proline for collagen synthesis[36]. Although alternative macrophage activation is induced by IL-4/13 production, Arg1-expressing macrophages can downregulate inflammation, suppress Th2 cytokine production and reduce tissue scarring and pathology[37]. In addition, the anti-inflammatory cytokine, IL-10, plays a central regulatory role in the pathogenesis of schistosomiasis, and the maintenance of IL-10 expression during acute and chronic schistosome infection is critical for host survival[38]. In this study, TLR2 was found to be the key PRR associated with the production of pro-inflammatory cytokines, IL-10, and Arg-1 expression from BMDMs stimulated with ES *in vitro*. Surprisingly, we observed a significantly higher mRNA levels of *IL-6*, *MCP-1* (*CCL2*), *IL-10*, and *Arg-1* in the liver macrophages of infected wild-type mice compared to that of TLR2^-/-^ mice, which was similar to M2b polarization *in vitro*. This finding suggests that ES antigens could induce the polarization of liver macrophages *in vivo*. However, the levels of *IL-6*, *MCP-1* (*CCL2*), *IL-10*, and *Arg-1* mRNA were found to be downregulated at 6 weeks post-infection. Thus, since similar serum levels of IL-4 and IL-13 were enhanced in both WT and TLR2 KO mice (S2 Fig), type II immunity may help to regulate levels of inflammatory cytokine mRNA in this study. Furthermore, the mRNA levels of *RELMa* were decreased in liver macrophages from TLR2 KO mice compared with that in WT mice at 4 weeks post-infection, which seemed different from that on stimulation with ES *in vitro*. Higher serum levels of IFN-γ that promoted M1 polarization and inhibited M2 polarization were observed in TLR2 KO mice compared with WT mice at 4 weeks post-infection (S2 Fig), thus type I immunity may help to downregulate the expression of *RELMa*. More importantly, IL-6 can recruit both neutrophils and monocytes during inflammation [39]. Moreover, MCP-1 accelerates liver fibrosis by promoting Ly-6C^+^ macrophage infiltration [40]. Our data indicate that TLR2 can increase the population of M2-type macrophages and neutrophil infiltration in the liver, perhaps because higher levels of MCP-1 (CCL2) and IL-6 are produced by ES-stimulated macrophages. Taken together, these data help explain the increased granuloma size and greater collagen deposition in the liver of wild-type mice. However, the mechanisms by which ES antigen-stimulated macrophages promote liver pathology require further investigation.

In summary, we have presented a detailed study of the molecular events that occur in BMDMs following exposure to ES antigens derived from eggs, which led to the production of pro-inflammatory cytokines and IL-10. This mechanism involves the activation of p65 downstream of TLR2 via the phosphorylation of p38, ERK1/2, and JNK1/2. Additionally, we showed that this mechanism is also responsible for the immunoregulatory activity in schistosomiasis, which provides an early *in vitro* demonstration of the role of ES Ags in modulating immunopathology downstream of TLR2. Finally, we observed that the production of IL-6, IL-10, MCP-1, and Arg-1 by macrophages in response to ES extends beyond an *in vitro* phenomenon and is also evident in the liver macrophages of WT-infected mice, which rapidly produce high levels of *IL-6*, *IL-10*, *MCP-1*, and *Arg-1* mRNA *in vivo* following egg deposition. This early and rapid release of IL-6, IL-10, MCP-1, and Arg-1 has the potential to greatly modulate the immune response to limit inflammation and tissue damage in the liver by conditioning the microenvironment. More importantly, M2 polarization dependent on enhanced TLR2 signaling would reduce the level of liver damage and promote fibrosis. While our data suggest that targeted TLR2 signaling inhibitors may have therapeutic effect during the acute phases of schistosomiasis, further study is still required to address the role of TLR2 signaling to better understand the potential benefits of its inhibitors in treatment of chronic disease and delineate novel insight into the immune interplay underlying schistosomiasis. For this reason, the results generated from this study might provide evidence supporting the necessity to include TLR2 signaling as a novel therapeutic target for schistosomiasis.

## Supporting information

S1 Table List of primers used for RT-qPCR analysis. (XLSX)

S1 Fig ES promoted M2b polarization in a TLR2-dependent manner. The levels of *IL-6*, *TNF-a*, *IL-10*, *IL-12*, *IL-1β*, *MCP-1*, *iNOS*, *Arg-1*, *RELMa*, and *Ym1* mRNA in BMDMs from both wild-type and TLR2 KO mice after stimulation with 1 μg/mL ES for 4 h were analyzed by RT-qPCR. The data shown are the results of a representative experiment from three independent experiments and were analyzed by two-way ANOVA.
(TIF)

S2 Fig Type I and type II cytokine profile in both infected wild-type and TLR2 KO mice. Serum levels of IFN-γ, IL-4, and IL-13 in both infected WT and TLR2 KO mice were detected by ELISA. Data, obtained from 10 mice per group, are analyzed by two-way ANOVA.
(TIF)
