# Supplementary figures and images for: Toll-like receptor-2 regulates macrophage polarization induced by excretory-secretory antigens from Schistosoma japonicum eggs and promotes liver pathology in murine schistosomiasis
Source: PLoS Negl Trop Dis. 2018 Dec 27;12(12):e0007000. doi: 10.1371/journal.pntd.0007000 (PMC6307705; doi:10.1371/journal.pntd.0007000)

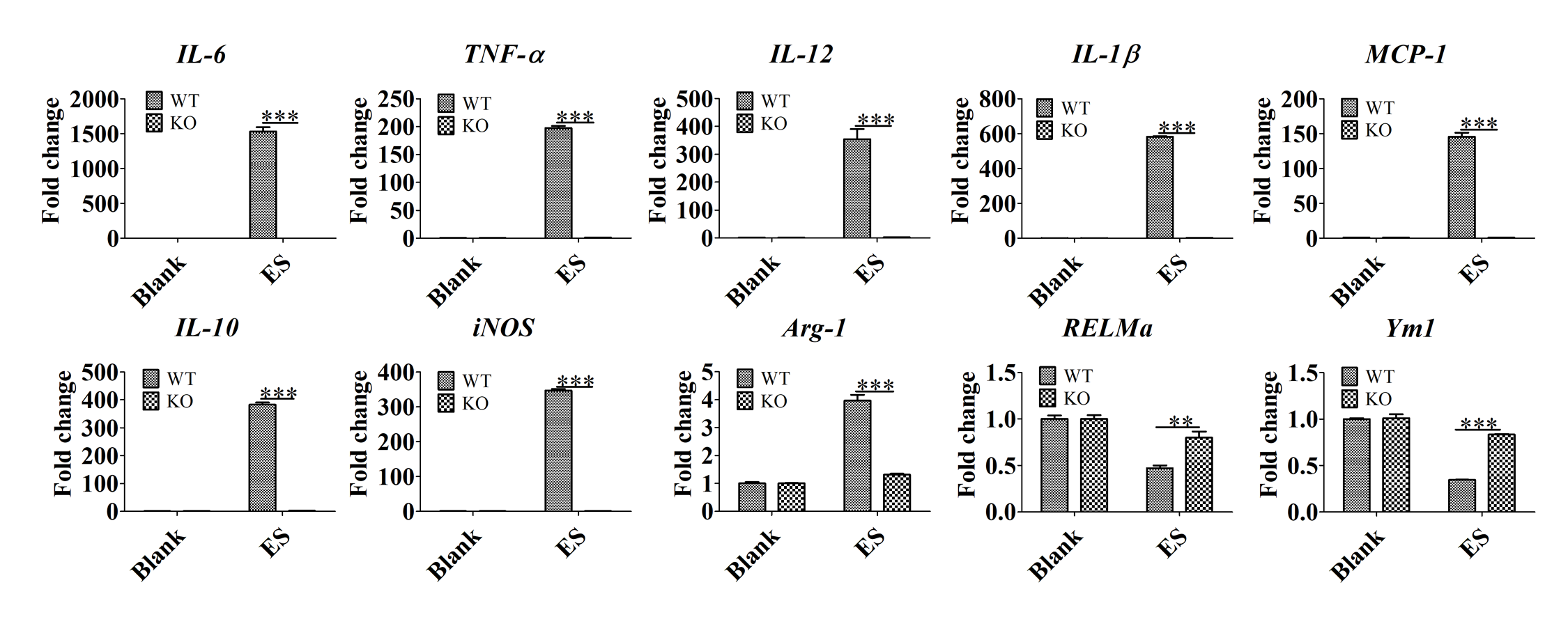

Supplement: S1 Fig — The levels of IL-6, TNF-a, IL-10, IL-12, IL-1β, MCP-1, iNOS, Arg-1, RELMa, and Ym1 mRNA in BMDMs from both wild-type and TLR2 KO mice after stimulation with 1 μg/mL ES for 4 h were analyzed by RT-qPCR. The data shown are the results of a representative experiment from three independent experiments and were analyzed by two-way ANOVA. (TIF) [file pntd.0007000.s002.tif]

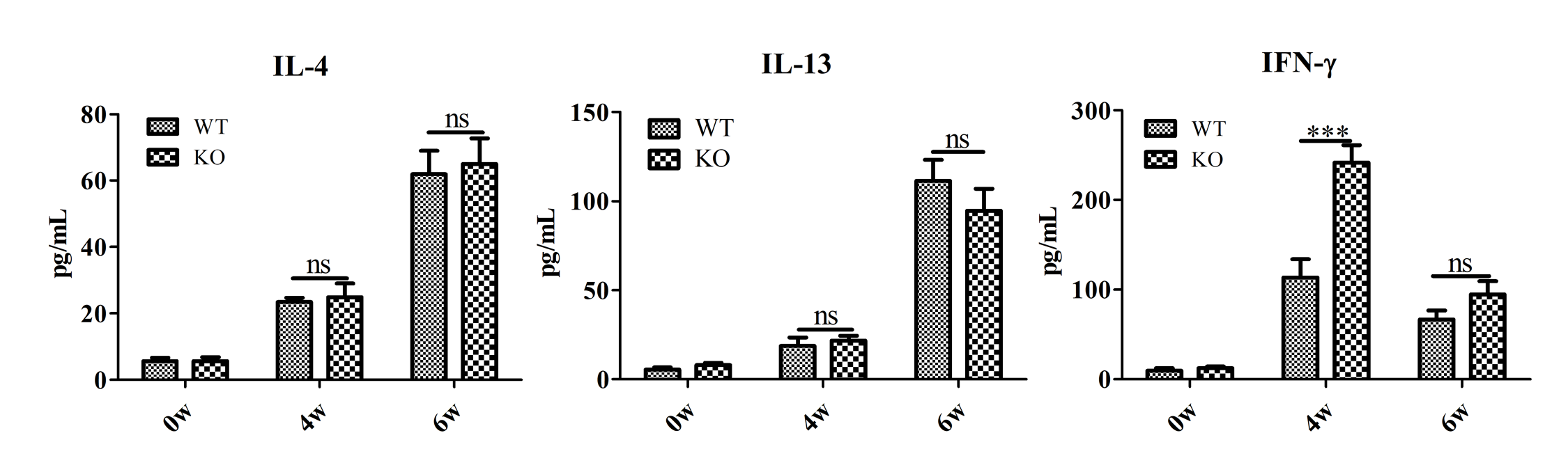

Supplement: S2 Fig — Serum levels of IFN-γ, IL-4, and IL-13 in both infected WT and TLR2 KO mice were detected by ELISA. Data, obtained from 10 mice per group, are analyzed by two-way ANOVA. (TIF) [file pntd.0007000.s003.tif]
